# Supplementary material for: Associations of SARS-CoV-2 antibodies with birth outcomes: Results from three urban birth cohorts in the NIH environmental influences on child health outcomes program
Source: PLoS One. 2023 Nov 22;18(11):e0293652. doi: 10.1371/journal.pone.0293652 (PMC10664934; doi:10.1371/journal.pone.0293652)
Supplement: S1 File — (DOCX) [file pone.0293652.s001.docx]

**Supplementary Tables: Associations of SARS-CoV-2 Antibodies with Birth Outcomes: Results from Three Urban Birth Cohorts in the NIH Environmental Influences on Child Health Outcomes Program**

|  | Negative serology to all antigens | Indeterminate to at least one antigen | IgM/G/A positive to at least one antigen |
| --- | --- | --- | --- |
| No self-report infection | 94 | 27 | 77 |
| Self-report infection | 11 | 2 | 0 |

**Supplement Table 1. Cross tabulations of serology against self-report of infection.**

**Supplement Table 2. Pearson Correlations of Stress and Infection and Serology in the Study Population.**

|  | past infection | combined serology | IgM status | IgG1 status | IgG2 status | IgG3 status | IgG4 status | IgA status | N combined serology | S1 combined serology | S2 combined serology | RBD combined serology | Stress scale |
| --- | --- | --- | --- | --- | --- | --- | --- | --- | --- | --- | --- | --- | --- |
|  |  |  |  |  |  |  |  |  |  |  |  |  |  |
| past infection | 1 |  |  |  |  |  |  |  |  |  |  |  |  |
| P value | N/A |  |  |  |  |  |  |  |  |  |  |  |  |
|  |  |  |  |  |  |  |  |  |  |  |  |  |  |
| combined serology | 0.0764 | 1 |  |  |  |  |  |  |  |  |  |  |  |
| P value | 0.2702 | N/A |  |  |  |  |  |  |  |  |  |  |  |
|  |  |  |  |  |  |  |  |  |  |  |  |  |  |
| IgM status | -0.0803 | 0.3765 | 1 |  |  |  |  |  |  |  |  |  |  |
| P value | 0.2465 | 0 | N/A |  |  |  |  |  |  |  |  |  |  |
|  |  |  |  |  |  |  |  |  |  |  |  |  |  |
| IgG1 status | 0.1713 | 0.704 | 0.2334 | 1 |  |  |  |  |  |  |  |  |  |
| P value | 0.0129 | 0 | 0.0006 | N/A |  |  |  |  |  |  |  |  |  |
|  |  |  |  |  |  |  |  |  |  |  |  |  |  |
| IgG2 status | 0.046 | 0.6616 | 0.1956 | 0.4909 | 1 |  |  |  |  |  |  |  |  |
| P value | 0.5075 | 0 | 0.0044 | 0 | N/A |  |  |  |  |  |  |  |  |
|  |  |  |  |  |  |  |  |  |  |  |  |  |  |
| IgG3 status | 0.0906 | 0.6707 | 0.2719 | 0.6044 | 0.5754 | 1 |  |  |  |  |  |  |  |
| P value | 0.1911 | 0 | 0.0001 | 0 | 0 | N/A |  |  |  |  |  |  |  |
|  |  |  |  |  |  |  |  |  |  |  |  |  |  |
| IgG4 status | 0.0359 | 0.423 | 0.0079 | 0.2718 | 0.5595 | 0.5354 | 1 |  |  |  |  |  |  |
| P value | 0.6054 | 0 | 0.9095 | 0.0001 | 0 | 0 | N/A |  |  |  |  |  |  |
|  |  |  |  |  |  |  |  |  |  |  |  |  |  |
| IgA status | 0.0773 | 0.6775 | 0.4046 | 0.5806 | 0.698 | 0.7185 | 0.531 | 1 |  |  |  |  |  |
| P value | 0.265 | 0 | 0 | 0 | 0 | 0 | 0 | N/A |  |  |  |  |  |
|  |  |  |  |  |  |  |  |  |  |  |  |  |  |
| N combined serology | 0.1558 | 0.4717 | 0.3083 | 0.4332 | 0.2426 | 0.3883 | 0.1749 | 0.3241 | 1 |  |  |  |  |
| P value | 0.024 | 0 | 0 | 0 | 0.0004 | 0 | 0.0109 | 0 | N/A |  |  |  |  |
|  |  |  |  |  |  |  |  |  |  |  |  |  |  |
| S1 combined serology | 0.1246 | 0.7838 | 0.4053 | 0.7249 | 0.6596 | 0.6897 | 0.4652 | 0.8169 | 0.5019 | 1 |  |  |  |
| P value | 0.0717 | 0 | 0 | 0 | 0 | 0 | 0 | 0 | 0 | N/A |  |  |  |
|  |  |  |  |  |  |  |  |  |  |  |  |  |  |
| S2 combined serology | 0.1023 | 0.7958 | 0.3136 | 0.6549 | 0.7188 | 0.7011 | 0.4593 | 0.6876 | 0.3854 | 0.7128 | 1 |  |  |
| P value | 0.1394 | 0 | 0 | 0 | 0 | 0 | 0 | 0 | 0 | 0 | N/A |  |  |
|  |  |  |  |  |  |  |  |  |  |  |  |  |  |
| RBD combined serology | 0.1378 | 0.7755 | 0.3621 | 0.7724 | 0.6815 | 0.685 | 0.4803 | 0.7658 | 0.5094 | 0.9246 | 0.707 | 1 |  |
| P value | 0.0461 | 0 | 0 | 0 | 0 | 0 | 0 | 0 | 0 | 0 | 0 | N/A |  |
|  |  |  |  |  |  |  |  |  |  |  |  |  |  |
| Stress scale | 0.0157 | -0.0893 | -0.1166 | -0.1702 | -0.0845 | -0.1723 | -0.1215 | -0.1511 | -0.0334 | -0.1824 | -0.0951 | -0.1693 | 1 |
| P value | 0.8641 | 0.3277 | 0.2007 | 0.0609 | 0.3547 | 0.0577 | 0.1824 | 0.0967 | 0.7149 | 0.0444 | 0.2977 | 0.0624 | N/A |

| **Supplement Table 3. Comparing Birth Outcomes in Mothers by Serology Subtype (N=211).** | | | | |  | |  | |  |
| --- | --- | --- | --- | --- | --- | --- | --- | --- | --- |
| IgM | Univariate | | | | Multivariable | | | |  |
|  | Indeterminate | | Seropositive/Self-Report Infected | | Indeterminate | | Seropositive/Self-Report Infected | |  |
| Gestational Age, weeks (95% CI) | -0.05 (-0.81, 0.72) | | -0.64 (-1.70, 0.41) | | 0.08 (-0.87, 0.71) | | -0.76 (-1.83, 0.31) | |  |
| Birth Weight, grams (95% CI) | 7.9 (-225, 240) | | -407 (-728, -85)* | | 66 (-168, 300) | | -434 (-752, -116)** | |  |
| Preterm Birth, OR (95% CI) | 1.30 (0.15, 11.0) | | 6.29 (1.12, 35.2)* | | 1.19 (0.12, 11.3) | | 8.75 (1.22, 62.4)* | |  |
| Low Birth Weight, OR (95% CI) | 1.75 (0.20, 15.4) | | 3.71 (0.40, 34.6) | | 1.51 (0.16, 14.4) | | 4.15 (0.40, 43.2) | |  |
| Weight for Gestational Age Z-score (95% CI) | 0.08 (-0.38, 0.55) | | -0.65 (-1.30, -0.002)* | | 0.13 (-0.33, 0.60) | | -0.73 (-1.36, -0.10)* | |  |
| Small for Gestational Age, OR (95% CI) | 2.18 (0.44, 10.8) | | + | | 3.78 (0.61, 23.4) | | + | |  |
|  |  | |  | |  | |  | |  |
| IgG | Univariate | | | | Multivariable | | | |  |
|  | Indeterminate | | Seropositive/Self-Report Infected | | Indeterminate | | Seropositive/Self-Report Infected | |  |
| Gestational Age, weeks (95% CI) | -0.17 (-0.86, 0.53) | | -0.12 (-0.59, 0.35) | | -0.27 (-0.98, 0.44) | | -0.21 (-0.68, 0.27) | |  |
| Birth Weight, grams (95% CI) | -117 (-331, 96) | | -1 (-144, 143) | | -95 (-309, 119) | | -10 (-154, 134) | |  |
| Preterm Birth, OR (95% CI) | 5.60 (1.29, 24.2)* | | 1.24 (0.27, 5.69) | | 7.76 (1.38, 51.6)* | | 1.32 (0.26, 6.71) | |  |
| Low Birth Weight, OR (95% CI) | 3.42 (0.54, 21.7) | | 1.66 (0.33, 8.47) | | 3.04 (0.44, 21.0) | | 1.57 (0.30, 8.38) | |  |
| Weight for Gestational Age Z-score (95% CI) | -0.17 (-0.60, 0.26) | | 0.04 (-0.25, 0.33) | | -0.29 (-0.80, 0.21) | | 0.01 (-0.32, 0.34) | |  |
| Small for Gestational Age, OR (95% CI) | 0.52 (0.06, 4.28) | | 0.34 (0.07, 1.64) | | + | | 1.02 (0.20, 5.34) | |  |
|  |  | |  | |  | |  | |  |
| IgA | Univariate | | | | Multivariable | | | |  |
|  | Indeterminate | | Seropositive/Self-Report Infected | | Indeterminate | | Seropositive/Self-Report Infected | |  |
| Gestational Age, weeks (95% CI) | 0.13 (-0.71, 0.97) | | -0.08 (-0.63, 0.46) | | 0.12 (-0.73, 0.98) | | -0.15 (-0.71, 0.40) | |  |
| Birth Weight, grams (95% CI) | -77 (-335, 181) | | 12 (-156, 180) | | -87 (-344, 169) | | -7 (-174, 161) | |  |
| Preterm Birth, OR (95% CI) | + | | 0.83 (0.17, 4.01) | | + | | 0.84 (0.16, 4.33) | |  |
| Low Birth Weight, OR (95% CI) | + | | 1.27 (0.25, 6.56) | | + | | 1.22 (0.22, 6.61) | |  |
| Weight for Gestational Age Z-score (95% CI) | -0.19 (-0.71, 0.33) | | 0.04 (-0.30, 0.38) | | -0.04 (-0.47, 0.38) | | 0.04 (-0.24, 0.33) | |  |
| Small for Gestational Age, OR (95% CI) | + | | 0.74 (0.16, 3.53) | | 0.45 (0.05, 4.15) | | 0.34 (0.07, 1.80) | |  |
| + All indeterminate mothers had children with normal BW and GA. | | | | | | | | |  |
| **Supplement Table 4. Comparing Birth Outcomes in Mothers by Antigen Response (N=211).** | | | | | | | | | |
| N | | Univariate | | | | Multivariable | | | |
|  | | Indeterminate | | Seropositive/Self-Report Infected | | Indeterminate | | Seropositive/Self-Report Infected | |
| Gestational Age, weeks (95% CI) | | 0.10 (-0.76, 0.96) | | -0.37 (-1.09, 0.34) | | 0.05 (-0.81, 0.93) | | -0.41 (-1.13, 0.32) | |
| Birth Weight, grams (95% CI) | | -211 (-475, 52) | | 23 (-195, 242) | | -213 (-474, 48) | | -9 (-227, 209) | |
| Preterm Birth, OR (95% CI) | | 3.50 (0.67, 18.3) | | 1.05 (0.12, 8.83) | | 3.88 (0.64, 23.7) | | 1.58 (0.17, 14.6) | |
| Low Birth Weight, OR (95% CI) | | 2.18 (0.24, 19.5) | | 1.42 (0.16, 12.4) | | 2.05 (0.22, 19.3) | | 1.65 (0.18, 15.1) | |
| Weight for Gestational Age Z-score (95% CI) | | -.57 (-1.10, -0.04)* | | 0.18 (-0.26, 0.61) | | -0.51 (-1.03, 0.002) | | 0.08 (-0.35, 0.51) | |
| Small for Gestational Age, OR (95% CI) | | + | | 0.75 (0.09, 6.12) | | + | | 1.22 (0.13, 11.1) | |
|  | |  | |  | |  | |  | |
| S1 | | Univariate | | | | Multivariable | | | |
|  | | Indeterminate | | Seropositive/Self-Report Infected | | Indeterminate | | Seropositive/Self-Report Infected | |
| Gestational Age, weeks (95% CI) | | 0.56 (-0.36, 1.49) | | -0.20 (-0.68, 0.29) | | 0.49 (-0.46, 1.44) | | -0.25 (-0.75, 0.24) | |
| Birth Weight, grams (95% CI) | | -3.65 (-290, 283) | | -51 (-200, 98) | | -1 (-288, 286) | | -78 (-228, 72) | |
| Preterm Birth, OR (95% CI) | | + | | 0.52 (0.11, 2.50) | | + | | 0.61 (0.12, 3.15) | |
| Low Birth Weight, OR (95% CI) | | + | | 0.80 (0.16, 4.10) | | + | | 0.81 (0.15, 4.39) | |
| Weight for Gestational Age Z-score (95% CI) | | -0.14 (-0.72, 0.44) | | -0.04 (-0.34, 0.26) | | -0.20 (-0.77, 0.37) | | -0.12 (-0.42, 0.17) | |
| Small for Gestational Age, OR (95% CI) | | + | | 0.47 (0.10, 2.20) | | + | | 0.73 (0.15, 3.71) | |
|  | |  | |  | |  | |  | |
| S2 | | Univariate | | | | Multivariable | | | |
|  | | Indeterminate | | Seropositive/Self-Report Infected | | Indeterminate | | Seropositive/Self-Report Infected | |
| Gestational Age, weeks (95% CI) | | -0.13 (-0.95, 0.68) | | -0.08 (-0.57, 0.42) | | -0.18 (-1.01, 0.65) | | -0.15 (-0.65, 0.36) | |
| Birth Weight, grams (95% CI) | | -117 (-369, 134) | | 22 (-129, 174) | | -95 (-346, 155) | | 3 (-149, 155) | |
| Preterm Birth, OR (95% CI) | | 1.10 (0.13, 9.41) | | 0.62 (0.13, 3.03) | | 1.24 (0.15, 14.0) | | 0.60 (0.12, 3.11) | |
| Low Birth Weight, OR (95% CI) | | 1.80 (0.20, 16.4) | | 1.02 (0.19, 5.41) | | 1.68 (0.17, 16.7) | | 1.01 (0.18, 5.49) | |
| Weight for Gestational Age Z-score (95% CI) | | -0.14 (-0.65, 0.37) | | 0.08 (-0.22, 0.39) | | -0.12 (-0.61, 0.37) | | 0.04 (-0.26, 0.34) | |
| Small for Gestational Age, OR (95% CI) | | 0.87 (0.01, 7.24) | | 0.24 (0.03, 1.93) | | 1.33 (0.14, 12.5) | | 0.27 (0.03, 2.29) | |
|  | |  | |  | |  | |  | |
| RBD | | Univariate | | | | Multivariable | | | |
|  | | Indeterminate | | Seropositive/Self-Report Infected | | Indeterminate | | Seropositive/Self-Report Infected | |
| Gestational Age, weeks (95% CI) | | 0.80 (-0.16, 1.75) | | -0.34 (-0.83, 0.14) | | 0.71 (-0.27, 1.69) | | -0.41 (-0.91, 0.09) | |
| Birth Weight, grams (95% CI) | | 20 (-279, 319) | | -41 (-191, 109) | | 69 (-229, 367) | | -83 (-235, 69) | |
| Preterm Birth, OR (95% CI) | | + | | 1.57 (0.36, 6.82) | | + | | 1.36 (0.31, 5.87) | |
| Low Birth Weight, OR (95% CI) | | + | | 0.96 (0.25, 3.77) | | + | | 1.77 (0.38, 8.28) | |
| Weight for Gestational Age Z-score (95% CI) | | -0.20 (-0.77, 0.37) | | -0.12 (-0.42, 0.17) | | -0.12 (-0.71, 0.47) | | -0.07 (-0.37, 0.23) | |
| Small for Gestational Age, OR (95% CI) | | + | | 0.73 (0.15, 3.71) | | 1.75 (0.17, 18.1) | | 0.40 (0.05, 3.39) | |
|  | |  | |  | |  | |  | |
