# Supplementary material for: Associations of SARS-CoV-2 antibodies with birth outcomes: Results from three urban birth cohorts in the NIH environmental influences on child health outcomes program
Source: PLoS One. 2023 Nov 22;18(11):e0293652. doi: 10.1371/journal.pone.0293652 (PMC10664934; doi:10.1371/journal.pone.0293652)
Supplement: Attachment — Submitted filename: response to reviewer comments.docx [file pone.0293652.s002.docx]

Faten Abdelaal Okda
Academic Editor
PLOS ONE

Thanks for the thoughtful reviewer comments regarding our manuscript.

We address the journal requirements, editorial comments and reviews below point-by-point.

We look forward to further consideration and acceptance by PLOS ONE.

Sincerely,

Leonardo Trasande, MD, MPP on behalf of the coauthors

Journal requirements:

When submitting your revision, we need you to address these additional requirements.

1. Please ensure that your manuscript meets PLOS ONE's style requirements, including those for file naming. The PLOS ONE style templates can be found at

<https://journals.plos.org/plosone/s/file?id=wjVg/PLOSOne_formatting_sample_main_body.pdf> and

<https://journals.plos.org/plosone/s/file?id=ba62/PLOSOne_formatting_sample_title_authors_affiliations.pdf>

*We have done as requested.*

2. We note that the grant information you provided in the ‘Funding Information’ and ‘Financial Disclosure’ sections do not match.

When you resubmit, please ensure that you provide the correct grant numbers for the awards you received for your study in the ‘Funding Information’ section.

*We have reviewed the manuscript, and these sections match. In addition, we have reviewed the FUnding Information in the PLOS ONE system to ensure alignment.*

3. We note that you have indicated that data from this study are available upon request. PLOS only allows data to be available upon request if there are legal or ethical restrictions on sharing data publicly. For more information on unacceptable data access restrictions, please see <http://journals.plos.org/plosone/s/data-availability#loc-unacceptable-data-access-restrictions>.

In your revised cover letter, please address the following prompts:

a) If there are ethical or legal restrictions on sharing a de-identified data set, please explain them in detail (e.g., data contain potentially sensitive information, data are owned by a third-party organization, etc.) and who has imposed them (e.g., an ethics committee). Please also provide contact information for a data access committee, ethics committee, or other institutional body to which data requests may be sent.

b) If there are no restrictions, please upload the minimal anonymized data set necessary to replicate your study findings as either Supporting Information files or to a stable, public repository and provide us with the relevant URLs, DOIs, or accession numbers. For a list of acceptable repositories, please see <http://journals.plos.org/plosone/s/data-availability#loc-recommended-repositories>.

We will update your Data Availability statement on your behalf to reflect the information you provide.

*Sorry – there seems to be a misunderstanding. The NIH ECHO Program has policies about sharing the data through the NICHD Data and Specimen Hub. We cannot provide data directly. Because the NIH funded the research, we must follow the legal requirement of our funder.*

4. PLOS requires an ORCID iD for the corresponding author in Editorial Manager on papers submitted after December 6th, 2016. Please ensure that you have an ORCID iD and that it is validated in Editorial Manager. To do this, go to ‘Update my Information’ (in the upper left-hand corner of the main menu), and click on the Fetch/Validate link next to the ORCID field. This will take you to the ORCID site and allow you to create a new iD or authenticate a pre-existing iD in Editorial Manager. Please see the following video for instructions on linking an ORCID iD to your Editorial Manager account: <https://www.youtube.com/watch?v=_xcclfuvtxQ>

*The ORCID iD for the author has been updated.*

5. One of the noted authors is a group or consortium [program collaborators for Environmental influences on Child Health Outcomes]. In addition to naming the author group, please list the individual authors and affiliations within this group in the acknowledgments section of your manuscript. Please also indicate clearly a lead author for this group along with a contact email address.

*We referred to the authors for ECHO in the Acknowledgment section. Institutions were also listed. There is not a lead author for this group.*

6. Your ethics statement should only appear in the Methods section of your manuscript. If your ethics statement is written in any section besides the Methods, please move it to the Methods section and delete it from any other section. Please ensure that your ethics statement is included in your manuscript, as the ethics statement entered into the online submission form will not be published alongside your manuscript.

*The ethics statement only appears in the revised Methods section.*

7. Please upload a copy of Supporting Information (Supplementary Table 1,2,3,4) which you refer to in your text on page 11 and 12.

*The Supplementary Tables are uploaded.*

Please review your reference list to ensure that it is complete and correct. If you have cited papers that have been retracted, please include the rationale for doing so in the manuscript text, or remove these references and replace them with relevant current references. Any changes to the reference list should be mentioned in the rebuttal letter that accompanies your revised manuscript. If you need to cite a retracted article, indicate the article’s retracted status in the References list and also include a citation and full reference for the retraction notice.

*We confirm the accuracy of the references.*


Reviewer #1: The manuscript presents an attempt to evaluate the relationships of SARS-CoV-2 serology, self-reported infection, and pandemic-related stress with birth outcomes in largely urban cohorts from three ECHO cohort centers.
The pregnancy outcomes are well-defined and include gestational age (GA), birth weight (BW), and BW Z-score for gestational age (as continuous variables. Categorical outcomes included prematurity,
low birth weight (LBW) and small for gestational age (SGA).
The analyses did not document any differences in gestational age, birth weight, PTB, or LBW. Nearly identical results were obtained when comparing birth outcomes by serology status. The only positive association was the finding that children with lower birth weight and BW Z-score for GA (were more likely to deliver preterm in univariate models.
The study is performed in an elegant way and I do not have any major questions regarding the methodology.

*Thank you for the kind words!*

The only point worth explaining is the way the final sample was identified. It is said that the study was nested within a subsample of mothers and newborns enrolled in three participating ECHO cohorts. Why three different cohorts were utilized? What were the criteria for inclusion and exclusion?

*This was a convenience sample of three cohorts which chose to participate as a supplementary funding opportunity. We have clarified this in the manuscript.*

Reviewer #2: Here the authors aim at understanding the effect that SARS-CoV-2 infection has during or before pregnancy. In an observational study including three urban cohorts that gave birth between January 2020 to September 2021 they analyzed samples from 211 mothers for the presence of IgG1, IgG2, IgG3, IgG4, IgM and IgA subclass antibodies against N, S1, S2 and RBD areas of the spike protein. Pre-pandemic samples (n=234) were used to define non-infected antibody levels, and levels between 3-6 SD above background were classed as indeterminate whereas over 6 SD were considered positive. All mother with at least one subclass over 6SD were considered positive, mothers with a least on subclass in the 3-6 SD as indeterminate, and all classes below 3SD as negative. Seropositivity was seen in 36% of mothers, while 43% reported that they had been infected. IgG1 total seropositivity as well as reactivity against N and RBD was modestly correlated with reported infection. Neither serology + self reported infection nor serology alone was correlated with gestational age, birth weight, pre-term birth or low birth weight. IgM positivity was scored in 9 mothers, and in this case a significant decrease in birth weight was observed. This could not be solely explained by a posttraumatic stress score calculated from the mothers. In addition, in children born from mothers before any vaccines was available, decreased birth weights was also seen children born to IgM positive mothers.

During COVID-19 infection it has been shown that IgM antibodies occur in serum at approximately the same time as IgG and IgA antibodies. Since IgM then decrease more rapidly the data indicate that recent (possibly during pregnancy) infections with COVID-19 can influence pregnancy outcomes, but the authors argue caution as this result is only based on very few cases (n=9).

Overall, the study is of interest although its main conclusion is based on very few samples. It is well written, the data is presented in a clear and careful way, and the results are discussed cautiously. Although the data will not overall change the general recommendation to try to avoid infection with SARS-CoV- 2 during pregnancy, it gives some further support to the recommendation.

Specific points

I think the material is big enough to not only discuss the potential correlation between IgM antibodies and pregnancy/birth related problems but also the lack of any correlation to IgG levels. This could suggest that it is unlikely that pre-pregnancy (or even early pregnancy) infections will not interfere with outcomes. I fully understand that it is hard to rule out that this could be due to early problems are not scored (i.e. problems conceiving or early spontaneous abortion), but at least late problems from previous infection are unlikely. I would suggest a short paragraph about this in the discussion.

*We have added the requested clarification.*

On line 234, the sentence “When we compared mothers by immunoglobulin subtype (Table 2c), the small number of IgM seropositive mothers in the sample (n=9) had children with 407 grams lower birth weight (95% CI: 85-728) and BW Z-score for GA (0.65 SD; 95% CI 0.002-1.30) were more likely to deliver preterm (OR 6.29, 95% CI 1.12-25.2) in univariate models.” Is very hard to understand – please reformulate.

*We have clarified as requested.*
